# Word problems: a review of linguistic and numerical factors contributing to their difficulty

**DOI:** 10.3389/fpsyg.2015.00348

**Published:** 2015-04-01

**Authors:** Gabriella Daroczy, Magdalena Wolska, Walt Detmar Meurers, Hans-Christoph Nuerk

**Affiliations:** ^1^Diagnostics and Cognitive Neuropsychology, Department of Psychology, Eberhard Karls Universität TübingenTübingen, Germany; ^2^LEAD Graduate School, Eberhard Karls Universität TübingenTübingen, Germany; ^3^Department of Linguistics, Eberhard Karls Universität TübingenTübingen, Germany; ^4^Knowledge Media Research CenterTübingen, Germany

**Keywords:** word problems, linguistics complexity, numerical complexity, text properties, difficulty

## Abstract

Word problems (WPs) belong to the most difficult and complex problem types that pupils encounter during their elementary-level mathematical development. In the classroom setting, they are often viewed as merely arithmetic tasks; however, recent research shows that a number of linguistic verbal components not directly related to arithmetic contribute greatly to their difficulty. In this review, we will distinguish three components of WP difficulty: (i) the linguistic complexity of the problem text itself, (ii) the numerical complexity of the arithmetic problem, and (iii) the relation between the linguistic and numerical complexity of a problem. We will discuss the impact of each of these factors on WP difficulty and motivate the need for a high degree of control in stimuli design for experiments that manipulate WP difficulty for a given age group.

## Word Problems

### Introduction

Word problems (WPs) are part of the school curriculum and are taught at all levels of education. In WPs, relevant information is presented in the form of a short narrative rather than in mathematical notation (Verschaffel et al., 2000). Sometimes WPs specifically encode a quantitative relation between objects (Boonen et al., 2013). Many children from kindergarten through adulthood have severe difficulties in solving WPs (Nesher and Teubal, 1975; Riley et al., 1983; Lewis and Mayer, 1987; Hegarty et al., 1992; Verschaffel et al., 1992). Both linguistic and numerical complexity contributes to the difficulty in solving WPs. However, researchers have so far often focused on the one or the other aspect, depending on which field they come from. Even within the respective fields, linguistics, and numerical cognition, some aspects have been studied extensively, while others have been (strangely) neglected. For instance, we will see that semantics and discourse structures have been frequently studied in the context of WP complexity, but systematic syntactic manipulations are scarce. As regards numerical cognition, number properties like parity and magnitude as well as the type of mathematical reasoning have often been studied, but the type and the form of operations (e.g., carry-over effects) have not been investigated thoroughly in WPs, although they play an important role in current numerical cognition research (Moeller et al., 2011; Nuerk et al., 2011, 2015).

In this review, as researchers from the field of linguistics and the field of numerical cognition we have collaborated to provide a systematic overview of linguistic and numerical aspects relevant to solving WPs as well as their interaction. To capture a broad range of relevant facets in the review, we extended our view of the relevant literature with systematic keyword searches in several databases (Web of Science, Ebsco, Google Scholar, ScienceDirect) including the following terms: WPs, story problems in combination with situational model, performance, consistency hypothesis, language processing, relational terminology, semantic influence, rewording, semantic cues, number size and type, working memory, text comprehension, computational errors, operations, position of unknown. In **Table 1**, we present selected linguistic, mathematical and general factors investigated in previous studies.

**Table 1:** Selected linguistic, mathematical, and general factors investigated in previous studies.

**Linguistic factors**	**Mathematical factors**	**General factors**
**Structure** Structural complexity of basic quantitative properties (e.g., Number of letters, word and sentence length, proportion of complex words) (Searle et al., 1974; Nesher, 1976; Lepik, 1990) Vocabulary level (e.g., polysemous words, prepositional phrases, passive voice, clause structure; Spanos et al., 1988; Abedi et al., 1997; Abedi and Lord, 2001; Shaftel et al., 2006; Martiniello, 2008) Question wording/placing (Cummins et al., 1988)	**Property of numbers** Single digit (Lean et al., 1990) Multi digit (Haghverdi et al., 2012) Type of number [e.g., fraction: (Raduan, 2010), decimal number: problem size, role of number (De Corte et al., 1988)] Number magnitude [e.g., range of number smaller than 100: (Nesher, 1976)]	**Skills and social aspects** Social consequences (Schley and Fujita, 2014) Learning disabilities (Kingsdorf and Krawec, 2014) Successful/unsuccessful problem solvers (Hegarty et al., 1995) Calculation/word problem (WP) difficulties (Powell and Fuchs, 2014) Children/adults (De Corte et al., 1990; Hegarty et al., 1992)
	
	**Required operation** Addition subtraction (De Corte and Verschaffel, 1987) Multiplication division (De Corte et al., 1988) Given number (De Corte et al., 1990; Vicente et al., 2007)	**Categorization** Semantic structure of arithmetic WPs (Riley et al., 1983) Algebra textbook frequency (Mayer, 1981) Standard/non-standard WP (Jimenez and Verschaffel, 2014)

**Semantics** Linguistics verbal cues (van der Schoot et al., 2009) Phrasing in cue words (LeBlanc and Weber-Russell, 1996)**** Conceptual rewording (Vicente et al., 2007) Semantic/Object relation (?) Presence of distractor (Muth, 1992)	**Mathematical solution strategy** Counting from larger number (De Corte and Verschaffel, 1987) Position of the unknown (Garcia et al., 2006) Arithmetic fact retrieval (Orrantia et al., 2010) Number combination (Fuchs et al., 2009) Situation/Mental arithmetic strategy (Brissiaud and Sander, 2010)	**Solution strategies** Algebra WP/arithmetic WP (Koedinger and Nathan, 2004) WP solving theory-models (Kintsch and Greeno, 1985) Translation strategies (Hegarty et al., 1995) Spatial/visual representation (Boonen et al., 2013) Situation model (Thevenot et al., 2007)
	
	**Relevance of information** (Terao et al., 2004) **Numerical processes and representation** (MacGregor and Price, 1999; Goebel et al., 2014)	**Other aspects** Pedagogical factors (Lean et al., 1990) Socio-mathematics (Reusser, 1988) Stereotypes thinking (Yee and Lee, 1997) Real-word knowledge (Verschaffel et al., 1997) Response mode (De Corte et al., 1988)

Consistency effect (Lewis and Mayer, 1987) e.g., revisited: (Pape, 2003)		Computer tutors (Nathan et al., 1992) Computer simulation (Dellarosa, 1986)

Basic linguistics influence on numerical cognition (Lachmair et al., 2014)		

Working memory (Swanson et al., 1993)		

### Individual Differences and Social Factors

Individual differences and social factors must be also considered in WP research (Fite, 2002). For example, in the PISA studies—often measured with WPs—, mathematics literacy is a commonly used notion (Stacey, 2012). “It is defined as an individual’s capacity to identify and understand the role that mathematics plays in the world, to make well-founded judgments and to use and engage with mathematics in ways that meet the needs of that” (OECD, 2010). Unsuccessful WP solvers can experience negative social health and life outcome (Schley and Fujita, 2014). Even beyond social consequences, numerous studies focused on individual differences and group differences, such as students with and without learning disabilities (Kingsdorf and Krawec, 2014), and children with and without developmental disabilities (Neef et al., 2003). Hegarty et al. (1995) distinguished domain-specific strategies that successful and unsuccessful problem solvers develop with practice and how these strategies account for individual differences in performance. Different students – e.g., individuals with calculation difficulty, or WP difficulty (Powell and Fuchs, 2014) – may struggle with different types of WPs. Besides domain-general capabilities like IQ, the role of domain specific knowledge and processes were investigated to get a complete account of problem solving, basic cognitive abilities; visual, reading skills, mathematical skills, and metacognitive abilities involved in the solution process. For example Boonen et al. (2014) and Oostermeijer et al. (2014) explored the role of spatial ability and reading comprehension in WP solving, since good WP solvers do not select numbers and relational keywords but create a visual representation (Boonen et al., 2013).

*Social factors* like schooling, teachers and peers also deserve consideration because the way of responding [e.g., De Corte et al. (1988)], the scoring criteria, the presence of illustrations next to the text [e.g., Berends and van Lieshout (2009)], or solution models used by the teachers influence WP performance considerably. School WPs also support stereotypical thinking: WPs do not resemble problems in real-world situations (Yee and Lee, 1997). In addition, there is a strong tendency among both students and teachers to exclude real-world knowledge from their WP solution (Verschaffel et al., 1997), which is consistent with the observation that the problem solving process is also influenced by social cognitive and epistemic behavior settings (Reusser, 1988). Linguistic and pedagogical factors also affect children’s understanding of arithmetic WPs (Lean et al., 1990). Students’ beliefs about what doing and knowing mathematics means are rather different from the ideals (Jimenez and Verschaffel, 2014) and shaped by “socio-mathematical norms.” Resulting differences in motivation seem to influence the strategies used to solve WPs (Gasco and Villarroel, 2014). In sum, both individual differences and social factors contribute to WP performance and deserve consideration.

### Subcategories of Word Problems and Solution Strategies

Several different types of WPs—e.g., in the underlying mathematical structure or solvability— are often presented intermixed in one study without acknowledging the problem type. This is problematic. Different types of WPs are presented for various student groups, in different schools or different age groups. For example, Swanson et al. (2013) investigated the role of strategy instruction and cognitive abilities on WP solving accuracy. The mathematical WPs they used were: addition, subtraction, and multiplication without any further description of the problem type. However, the available literature has already shown that different categories of WPs may lead to different solution strategies and different error types. For instance, different semantic problem types result in different errors (Vicente et al., 2007) and have a different difficulty level (LeBlanc and Weber-Russell, 1996). Obviously, different scientific studies reporting results for different student or age groups cannot be easily compared to one another when they use different WP types; it cannot be determined whether differences should be attributed to group or study manipulation or differences in the used stimulus material. In the following, we outline the major distinctions discussed in the literature. Besides the difficulty level, WPs have been categorized with regard to various other attributes. Based on standard algebra text books, Mayer (1981) categorized WPs according to their frequency. Riley et al. (1983) created four groups based on the *semantic structure* of additive arithmetic WPs (change, compare, combine, equalize) and 18 further subcategories. For instance, the change problem –where there is a start, a change, and a result state –can be subdivided into three subcategories depending on which state is the unknown.

The *mathematical content* of WPs can also serve as a basis for categorization. Algebra WPs typically require translation into a mathematical formula, whereas arithmetic WPs are solvable with simple arithmetic or even mental calculation. In contrast to arithmetic WPs, algebraic reasoning WPs share the same numerals and signs (Powell and Fuchs, 2014) and the manipulation of those numbers and signals differs based on the question or expected outcome (Kieran, 1990). However, the distinction is not that straightforward, as in some cases both methods can be applied. For instance, in a study by Van Dooren et al. (2002), future secondary school teachers preferred the use of algebra even when an arithmetical solution seemed more evident, and some future primary school teachers rather applied arithmetical methods. Computer-aided environments have been introduced for algebraic WPs (Reusser, 1993) to support learning on “getting the formalism” and the “equation” (Nathan et al., 1992) and to allow students to generate, manipulate, and understand abstract formal expressions for WPs. However, solution approaches are not easily dissociable between arithmetic and algebraic problems. If a WP is intended to be solved with an equation, in some cases a simple arithmetic approach is enough (Gasco et al., 2014). Under some circumstances, it is even easier to solve WPs via alternative arithmetic strategies than by deriving algebraic equations. US children perform better on a story problem if it is in a money context and the numbers involve multiples of 25 (Koedinger and Nathan, 2004). While the distinction between algebra and arithmetic WPs is important for investigation and evaluation, in this review we concentrate mainly on arithmetic WPs.

*Standardized phrases* and the *idea that every problem is solvable* are other important attributes of many, but not all WPs. Textbooks generally suggest implicitly that every WP is solvable and that every numerical information is relevant (Pape, 2003). They usually provide standardized phrases and keywords that are highly correlated with correct solutions (Hinsley et al., 1977; ?). There are so-called non-standard WPs (Jimenez and Verschaffel, 2014) which can be non-solvable WPs or if they are solvable some have multiple solutions and may contain irrelevant data. In the recent literature, non-standard WPs are getting more and more attention (Yeap et al., 2005; Csikos et al., 2011). Children give a high level of incorrect answers to non-standard WPS because these seem to contradict their mathematics-related beliefs learned in the classroom. Reusser (1988) presented 97 first and second graders with the following sentence: “There are 26 sheep and 10 goats on a ship. How old is the captain?” and 76 students “solved” the problem using the numbers in the task. The rationale behind such studies is that always-solvable textbook problems with standardized phrases and including only relevant numerical information are hardly ecologically valid. Real-life WPs are not standardized, contain irrelevant information, and a solution may not always exist.

The above subcategories, which essentially characterize specific sets of WP properties, have a direct impact on human performance in WP. For space limitations, we cannot discuss the impact of all subcategories in detail, but we illustrate their impact on performance and strategies with two examples: (i) different subcategories can result in different errors, and involve different representations and processes. For example, a familiar misconception is that multiplication (Vergnaud, 2009) always makes the result larger (which is not true for *n* < 1), that division makes the results smaller, and that division always involves division of the larger number by the smaller, (ii) addition problems are strongly influenced (De Corte and Verschaffel, 1987) by the semantic structure (change, compare, combine). Carpenter et al. (1981) reported that the dominant factor in determining the children’s solution strategy was this semantic structure. For instance, *Change* problems [cf. the classification of Riley et al. (1983)] require the child to find the difference between the two numbers given in the problem; their nature influences the strategies children adopt. Riley et al. (1983) illustrates this with the following examples: Change 2: “Joe had eight marbles. Then he gave five marbles to Tom. How many marbles does Joe have now?” Change 3: “Joe had three marbles. Then Tom gave him some more marbles. Now Joe has eight marbles. How many marbles did Tom give him?” Almost all the children used a subtraction strategy (e.g., counting up) to solve Change 2. For Change 3 almost all the children used an addition strategy (e.g., counting down). In sum, the subcategories introduced in this section influence both performance and the choice of solution strategies.

Indeed, solution strategies have systematically been in the focus of WP research and addressed the following questions: how do children and adults solve WPs? Why do they make different errors and at which level of the solution process they do so? Which kind of semantic representation do they create of the WP? Which skills are necessary for the solution process? The first theories on WP solution processes (Kintsch and Greeno, 1985) have drawn on the text comprehension theories of Mayer (1982) and Van Dijk and Kintsch (1983). When solving problems, the solver first integrates the textual information into an appropriate situation model or a mental representation of the situation being described in the problem, which then forms the basis for a solution strategy. This approach was further applied by (Thevenot and Oakhill, 2005; Jimenez and Verschaffel, 2014; Kingsdorf and Krawec, 2014). An important foundation of those approaches is that solving WPs is not a simple translation of problem sentences into equations (Paige and Simon, 1966). Often both WPs and the corresponding numerical problems are done without language translation (Schley and Fujita, 2014). Several researchers have focused on abstraction as a reductive process involved in the translation process in the WPs. Nathan et al. (1992) argue that WPs solving is an exercise in text processing required for understanding the problem (Cummins et al., 1988), which is highly dependent upon language comprehension skills. Successfully solving WPs has been argued to require at least three distinct processes (Nesher and Teubal, 1975): (i) understanding and constructing the relation between text and arithmetic task, (ii) linguistic understanding of the WP itself, and (iii) solving the arithmetic tasks. Typically only the latter process is assumed to be shared with common arithmetic tasks. Many students can successfully solve common arithmetic tasks and they show good text comprehension skills. Yet they fail to solve WPs correctly. This suggests that other factors like solution strategies and building up a mental model of the task also play a major role for the WP performance.

## Linguistic Complexity and Linguistic Studies

In linguistics, the notion of complexity is discussed under a range of perspectives, with particularly fruitful definitions grounded in research on language evolution (Nichols, 1990) and language acquisition (Bulté and Housen, 2012). Following the latter, it is useful to delineate linguistic complexity from propositional complexity (the amount of meaning to be expressed) and discourse-interactional complexity (the interaction of participants in discourse). This makes it possible to zoom in on linguistic complexity as the degree to which a text at hand is elaborated and varied (Ellis, 2003, p. 340). Linguistic complexity can be analyzed with respect to all aspects of the linguistic system: from the words and their lexical and morphological aspects, via the way these words can be combined in syntax to form sentences, to the text structure, and overall discourse. Languages differ with respect to where in the linguistic system complexification is supported. For example, English makes use of word order to encode grammatical functions, whereas agglutinative languages such as Hungarian or Turkish make use of a rich morphological inventory for this and other uses. The implication of linguistic encoding differences is twofold: first, the difficulty of WPs is language-specific, thus linguistic manipulation leading to increased WP complexity in one language may not have an effect in another, more complex language. Second, the performance of language learners on WPs presented in a foreign language may be affected by the differences between the learner’s mother tongue and the language of the problem presentation. In the following two sections, we briefly summarize the main findings on aspects of linguistic complexity that affect performance.

### Structural Factors

Studies on the relation between linguistic structure and student performance on WPs have considered complexity at the micro-level of word and sentence forms as well as at the macro-level of the discourse structure of the WP passage. Early approaches addressed structural complexity in terms of basic quantitative properties of the WP text, such as the number of letters, words, sentences, mean word, and sentence length, or the proportion of complex (long) words (Searle et al., 1974; Nesher, 1976; Lepik, 1990). More linguistically motivated variables have been investigated in the context of comprehension difficulties in WPs for language learners, for the most part learners of English. At the vocabulary level, comprehension difficulties which result in problem solving difficulties for English language learners may stem from the presence of unfamiliar (low-frequency) words, polysemous words, idiomatic or culturally specific lexical references. At the sentence structure level, factors that have been shown to play a role include noun phrase length, the number of prepositional phrases and participial modifiers, the presence of passive voice and complex clause structure such as relative, subordinate, complement, adverbial, or conditional clauses (Spanos et al., 1988; Abedi et al., 1997; Abedi and Lord, 2001; Shaftel et al., 2006; Thevenot et al., 2007; Martiniello, 2008).

At the discourse structure level, specifically in terms of discourse ordering, the correspondence between the order in which numerical data is presented in the WP and the order in which it can be used to solve it has been shown to be a major predictive variable. Order-consistent problems result in better performance (Searle et al., 1974). Better performance has also been observed for simpler question wording or placing the question before the text results (Cummins et al., 1988).

### Semantic Factors

A single factor that is straightforwardly related to WP difficulty and that has been widely investigated is the presence or absence of explicit verbal cues whose semantics hint at the expected operation and thus directly lead toward the solution. Verbal cues include words and phrases of different categories: conjunctions (“and” for addition), adverbs (“left,” “more than,” “less than” for subtraction), or determiners (“each” for multiplication). Eye tracking studies have shown that subjects tend to focus on linguistic verbal cues and perform translation directly to the mathematical operation (e.g., Hegarty et al., 1992; van der Schoot et al., 2009).

Because verbal cues so often lead to default mathematical interpretation (Nesher, 1976), even small differences in phrasing in cue words can cause significant changes in performance (LeBlanc and Weber-Russell, 1996). This is especially relevant for young children (Lean et al., 1990), who in the course of development connect words such as “join,” “add,” “get,” “find,” or “take away” with concepts such as *putting together*, *separating*, *giving away*, or *losing*. A problem can thus be reworded by adding verbal clues which make the semantic relations more salient so that the underlying mathematical relation is more explicit. For example, the WP “There are five marbles. Two of them belong to Mary. How many belong to John?” can be reworded as “There are five marbles. Two of them belong to Mary. The rest belong to John. How many belong to John?” [from Cummins (1991)]. This kind of conceptual rewording has been shown to be useful to improve children’s performance on WPs (Vicente et al., 2007). Thus changes in wording can influence representation (De Corte et al., 1985).

Semantic or object relations between the objects described in the problem also relate to difficulty. Division problems usually involve functionally related objects (e.g., *tulips*–*vases*) and rarely categorically related objects (e.g., *tulips*–*daisies*; ?). By contrast, addition for the most part involves categorically related objects. The correlation between object relations and mathematical operations has been argued to reflect a structural correspondence between semantic and mathematical relations (Bassok et al., 1998). For this reason, the semantic structure properties of a WP have been emphasized as a more important factor contributing to difficulty than the syntactic structure (Yeap and Kaur, 2001; ?). Interestingly, an effect related to information load has been observed; the presence of content irrelevant to the core solution, i.e., the presence of numerical or linguistic distractors, results in higher error rates (Muth, 1992). De Corte and Verschaffel (1987) found that the semantic structure of WPs influences children’s choice of mathematical solution strategy. In terms of the broader task context, the required or expected way of responding to the WP has a big influence, especially for the domain of multiplication and division with rational numbers as argued in De Corte et al. (1988); for example, whether students are expected to answer the problem numerically or if they only have to indicate the required operation, or whether they respond in an open way or with multiple choice.

## Numerical Complexity and Numerical Studies

Arithmetic WPs have to be usually transformed mentally into an arithmetic problem and usually require an arithmetic solution (?). This means transforming word and numbers into the appropriate operation (Neef et al., 2003). Since the arithmetic problem has to be solved in the end, numerical representations and arithmetic processes will also play an important role in the solution process. In numerical cognition, different models and representations have been proposed (e.g., Dehaene and Cohen, 1995; Nuerk et al., 2011). However, the problem here is that the literature on WP often seems (with some exceptions) to be largely in a parallel research universe to the literature on numerical cognition and arithmetic processes, so that standard models of numerical cognition are hard to apply on the existing literature. What is more, WP research on numerical factors is also affected by the scoring criteria; in some studies on WP solving, computational errors are neglected, because in many studies researchers consider a solution as correct as long as the solver has chosen the correct mathematical model (Verschaffel and De Corte, 1990). This is not the case in behavioral numerical cognition research, where the correct result is usually essential and RTs, accuracies, error types, and solution types are analyzed based on the arithmetic problem and result.

**FIGURE 1:**
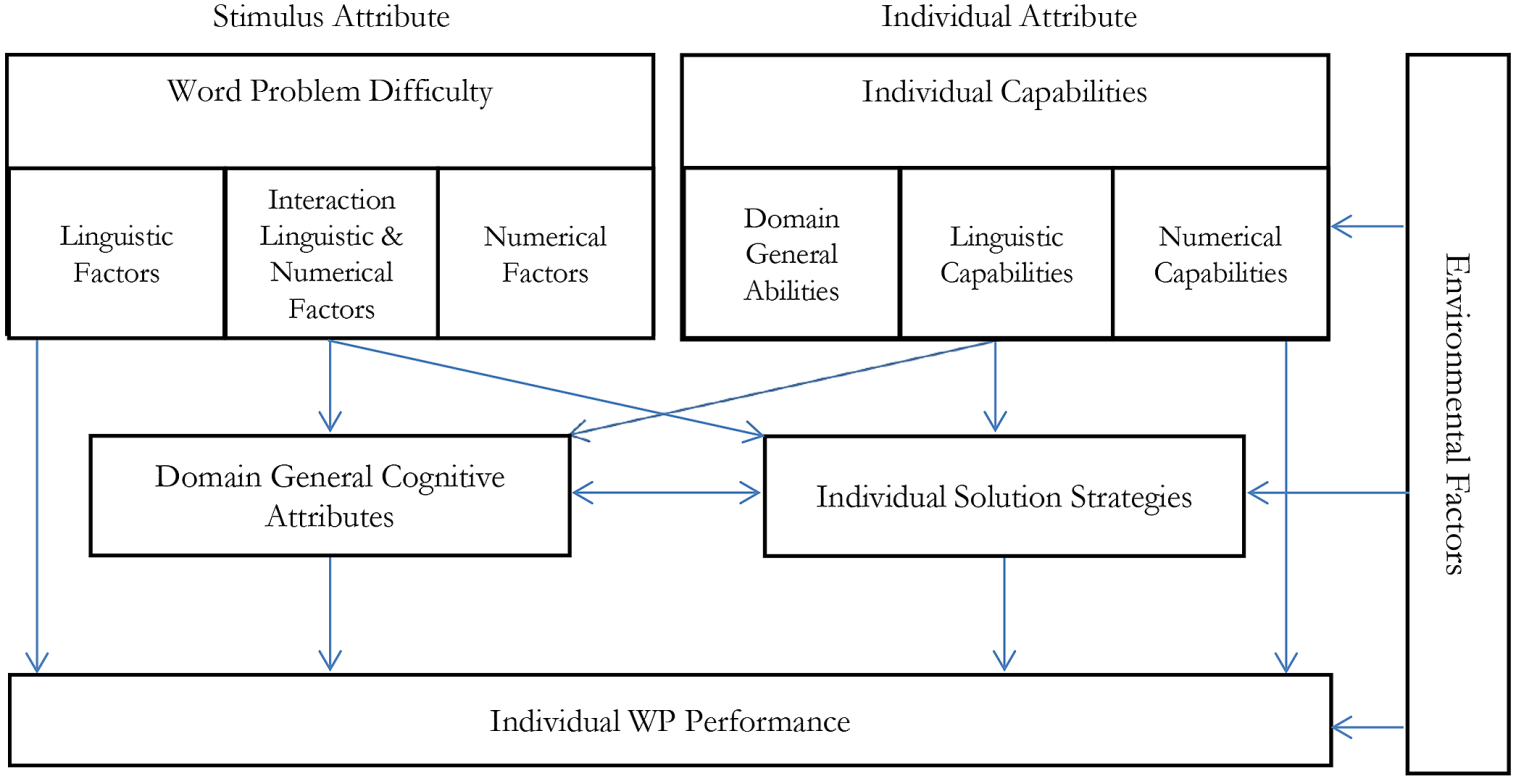
**This figure describes a possible theoretical process model of world problem solving based on this article and dissociating numerical and linguistic factors: Three general aspects are distinguished for predicting individual WP performance.** Stimulus Attributes (WP difficulty), individual attributes (capabilities), and environmental factors (e.g., teaching). WP difficulty comprises linguistic factors (such as linguistic complexity of the WP text, Section 2 of this article), numerical factors (such as numerical difficulty of the numerical problem, Section 3), and their interaction (such as the relation between text and arithmetic problem, Section 4). Individual Capabilities can refer to linguistic and numerical capabilities and domain- general abilities such as individual working memory capacity. Stimulus attributes and individual attributes influence individual WP performance both directly and over two mediator variables. One mediator variable refers to domain-general attributes, such as cognitive load. Complex linguistic and numerical stimulus attributes can increase cognitive load and the impact of increased complexity may be overadditive, especially when the joint linguistic and numerical complexity exceeds the cognitive load of an individual. On the other hand, those domain- general attributes are influenced by individual capability. Cognitive load for an individual with high linguistic or numerical abilities may be lower for the same problem than for an individual with low linguistic or numerical abilities. The second mediator variable refers to specific solution strategies. If specific solution strategies can be applied to a particular WP problem, because the problem type allows this and because the individual knows the strategy, solution strategies can facilitate WP solving. Finally, environmental factors (e.g.: teaching, scoring system… etc.) influence individual capabilities, solution strategies, and also directly individual WP Performance.

Numerical complexity can influence WP performance via at least three routes (see **Figure 1**):

(1) Direct route: WPs with more complex arithmetic structure are more difficult independent of linguistic complexity.
(2) Cognitive load: more complex arithmetic problems involve a higher cognitive load. For instance, carry problems are supposed to require more working memory resources. If the linguistic properties are also complex and the built-up of a mental model also requires more working memory resources, high arithmetic, and linguistic complexities could lead to over additive difficulties which could neither be explained by main effects of linguistic or numerical difficulty.
(3) Solution strategies: multi-digit numbers are harder to process than single-digit numbers (Nuerk et al., 2011; for a review) and arithmetic complexity usually increases with numbers of digits. Thevenot and Oakhill (2005) compared the influence of processing three-digit numbers and two-digit numbers on WP solution strategies. They showed that processing numerically more complex three-digit numbers facilitated alternative strategies by the participants. The authors suggested that higher work load and working memory led to this facilitation. For our review and the model in the revised manuscript, the important point is that they resort to less effortful strategies. Similar results were observed by Brissiaud and Sander (2010), who manipulated the size and order of the numbers and operation thus resulting in two different solution strategies: (i) situation-strategy WPs that are easy to solve with informal strategies, e.g., double-counting, derived number fact, or trial-error strategy, (ii) mental arithmetic-strategy WPs are “easy to solve with mental calculation, but only when the relevant arithmetic knowledge is used.” Number magnitude and order determined which strategy was used most likely. In this review, we suggest that resorting to alternative easier strategies is not restricted to number magnitude, but could be used with any numerical variable that allows simpler solutions. For instance, if a number bisection task were used in a text problem, we would also suggest that participants resort to easier strategies (e.g., checking the parities of the outer number), when the bisection problem gets more complex (e.g., larger interval, decade crossing etc.).

Nevertheless, some distinctions of numerical processes can be made in our review of the WP literature and are therefore proposed as an initial step in this review. Note that in our view this is not the end of the integration of numerical cognitive research and WP research, but rather just a beginning. For an overview of the investigation of specific numerical processes in current WP research, we suggest categorizing them into five categories:

(i) the property of numbers (parity, single digit/multi digit, problem size, ties, type of number, role of the number, number magnitude),
(ii) required operation (type, number)
(iii) mathematical solution strategies (larger number, place, automatic fact retrieval, position of the unknown),
(iv) relevance of the information.
(v) other numerical processes and representations

### Number Properties

While some studies have shown an effect of numerical complexity, from a numerical cognition view it is surprising that actually the arithmetic complexity has rarely been systematically considered as an isolated factor in WPs, although it is frequently examined in other arithmetic problems or simply the description of numbers is missing, e.g., De Corte et al. (1990). For instance,* parity* attributes are rarely considered in WPs, although in children it influences task performance and strategy choice in arithmetic tasks. For instance, in the number bisection task (Is the middle number *Y* the exact mean of *X* and *Z* in *X_Y_Z*?), parity influences performance. Trials with unequal parities of *X* and *Z* are easier to solve than trials with equal parities (Nuerk et al., 2002). We suggested that this is due to a change in strategy. In trials with unequal parity (e.g., 21_25_28), it is impossible that the middle number is the mean, because the mean of numbers with unequal parity is not an integer number (and only integers were used in the experiment). Therefore, participants may change their strategy after they discovered unequal parities and may not compute further to find out whether the middle number is really the mean. A later fMRI study (Wood et al., 2008) corroborated this assumption. In the easier unequal parity (“impossible”) condition, we observed more activation in the right ventrolateral prefrontal cortex, which is activated in cognitive set changes or when participants generate alternative solutions for a task. Thus, parity can influence performance and solution strategies in arithmetic. This seems not only the case in the bisection task, which is to our knowledge rarely used in WP research, but also in standard operations like addition and subtraction. A review by Hines (2013) suggests that parity influences the difficulty of addition and subtraction, but not multiplication, and tasks containing odd numbers are more difficult than with even ones. Such parity effects have received little attention in WP research so far. Furthermore, it seems that most WPs, especially for children, contain *single-digit numbers*; e.g., each answer was in the range of 1–9, e.g., in Lean et al. (1990), or Powell and Fuchs (2014), only few use *multi-digit numbers* (Haghverdi et al., 2012). In Nesher (1976) the range of numbers is smaller than 100, contained division two-digit numbers into one-digit number.

Explanations why the studies have chosen specific numbers, e.g., mentioning problem size, are rare. De Corte et al. (1990) and Orrantia et al. (2010) controlled for the number of sentences; the size of the numbers given in the problems. In the study of van der Schoot et al. (2009) the final answers were between 14 and 40, included no fraction, no negative number, no numerical value twice, and none of the possible answers resulted in another. However, different types of numbers were presented in WPs in some more studies: (i) fraction (Raduan, 2010), (ii) whole number, (iii) decimal number (Haghverdi et al., 2012); and their effect has been rarely investigated. Koedinger and Nathan (2004) found an effect for decimal numbers: “however we also observed a smaller situation facilitation effect whereby story performance was better than word equation performance under certain conditions: namely dealing with decimal numbers.”

The mixed use of single- and multi-digit numbers is problematic because in the last 15 years, numerous numerical cognition studies have shown that single-digit number processing cannot easily be generalized to multi-digit number processing, e.g., Nuerk et al. (2001); for reviews see Nuerk and Willmes (2005) and Nuerk et al. (2015). Nuerk et al. (2015) have identified 17 numerical effects linked to different numerical representation, which are specific for multi-digit number processing and which cannot be explained by single-digit number representations. Also even the same effects are different for single- and multi-digit numbers. For instance, Ashkenazi et al. (2009) have shown that the distance effect for two-digit numbers differentiates between dyscalculic and typically developing children. The sometimes seemingly arbitrary mix of single-digit and multi-digit number use in WP research is therefore not reasonable in our view given the state of numerical cognition research and the major differences between processing those different number types. The *role of a number* within an operation also influences WP complexity (De Corte et al., 1988). For example, in the case of addition the role means: addend, minuend or by multiplication: multiplicand, multiplier. One important finding from recent research on multiplication WPs is that children’s performances are strongly affected by the nature of the multiplier whether, e.g., it is an integer, decimal larger than 1 or a decimal smaller than 1. On the other hand, the size of the multiplicand has little or no effect on problem difficulty. De Corte et al. (1988) stated that “two multiplication problems with the same mathematical, semantic, and surface structure but different in terms of the nature of the given numbers can elicit very distinct levels of problems difficulty.” Indeed, this corresponds to recent findings that relatedness and consistency heavily influence the ease with which a multiplication problem can be solved cf. for relatedness (Domahs et al., 2006, 2007) and for consistency Verguts and Fias (2005).

Despite the major role of number properties in numerical cognition, number property has not been investigated extensively in the WPs (Fuchs et al., 2009). Nevertheless, numbers seem to play a major role. For instance, De Corte and Verschaffel (1986) observed that in their eye tracking study there was a relatively strong focus on the numbers in the problem. Twenty-five percent of the total solution time was spent in the two small number areas. However, major number properties of numerical cognitions research such as number magnitude are rarely systematically considered in WP research. In our view, more dialog between fields, – numerical cognition and WP research – seems necessary.

### Required Operation

Carrying out operations are necessary steps in solving arithmetic WPs. Operations have been used extensively in WPs. Most errors seem to originate from people’s failure to understand the language of WPs, i.e., the linguistic embedding of the calculation problem (Schumacher and Fuchs, 2012), and arithmetic computation errors themselves (Raduan, 2010; Kingsdorf and Krawec, 2014). Some errors may result from correct calculation performed on incorrect problem representation (Lewis and Mayer, 1987) and different operations may lead to different solution strategies. The most usual operation used in WP experiments are addition and subtraction (Carpenter et al., 1984; De Corte et al., 1988; Schumacher and Fuchs, 2012). Even the classification of Riley et al. (1983) was made for elementary addition and subtraction. Research in the 1980s and 1990s concentrated on how children learn to do one step addition and subtraction problems involving small whole numbers; see the review from Vicente et al. (2007). Later, the focus was more on the multiplication WPs or mixed WPs – e.g., Swanson (2004). Greer (1992) presented a framework categorization of multiplication and division WPs on the basis of the types of quantities involved (positive integers, fraction, and decimals) as models of situation. The semantic problem structure also influences the solution strategies for addition and subtraction.

Choosing the correct operation strongly depends on the type of the given numbers in the problem (De Corte et al., 1990). As already shortly outlined in above subsection on problem types, there is a huge body of research on what makes addition, subtraction, or multiplication problems difficult. Carry operations (e.g., 28 + 47; the decade value 1 from the unit sum 15 has to be carried over to the decade sum) have long been known to make multi-digit addition more difficult in children and adults; see Nuerk et al. (2015) for a review. However, solution strategies differ between children and adults – eye movement data suggest that in a choice reaction task elementary school children always compute and search for the correct results, while adults seem to also decide based on the rejection of the incorrect result. What is more, even within the carry operations at least three different cognitive processes can be identified for adults: unit sum calculation, carry detection, and carry execution (Moeller et al., 2011). Inability to execute one of these processes may lead to worse performance in carry problems in particular. Carry addition problems seem to require larger working memory resources (Ashcraft, 1995; Furst and Hitch, 2000). If cognitive load/working memory demand is high, because both the linguistic and the numerical complexity of the WP are large, this may lead to over additive problems in the domain-general processing stages involved in WP solving — see **Figure 1**, for an elaboration. For multiplication, we know that relatedness, ties, whether a problem stems from the 0, 1, 2, 5, or 10 row (Josta et al., 2009), or consistency influence the difficulty of a multiplication problem (Domahs et al., 2006, 2007). Although such factors have been extensively studied in numerical cognition research, they are – to the best of our knowledge – rarely considered in WP research. Since we know that these factors make the arithmetic computation, which is part of the WP solution, this lack of consideration is again problematic in our view.

### Mathematical Solution Strategies

Mathematical solution strategy variations have been studied extensively, and can be a function of linguistic factors like wording, semantic categories and propositions. However, how individuals come up with mathematical solution strategies can be also be influenced by numerical factors like number magnitude (Thevenot and Oakhill, 2005). Such variables, which are independent of other factors, make WPs harder and/or influence numerical representations, have rarely been studied. The position/place of the unknown variable has an effect on representation (Garcia et al., 2006). Even studies about working memory also investigated the position of the unknown variable (Swanson, 2004). The strategy of counting on from larger is easier if the bigger number is represented first (Wilkins et al., 2001). Even for adults: 4 + 2 = 6, and 2 + 4 = 6, which are mathematically equivalent, may psychologically imply different meanings (Kaput, 1979). The sequence of the numbers, e.g., whether a problem starts with the smaller or with the larger number (Verschaffel and De Corte, 1990), the position of the numbers and particular words (Schumacher and Fuchs, 2012) influence children’s solution of elementary addition and subtraction problems. For example, in change problems children typically look for a specific number to begin with, depending on task features, like the first mentioned number (Lean et al., 1990; Wilkins et al., 2001), the type of problem (start or change set), and the size of the numbers (Verschaffel and De Corte, 1990).

Arithmetic fact retrieval is a well researched ubiquitous strategy in numerical cognition but less so in the domain of WPs. Orrantia et al. (2010) found that arithmetic fact retrieval is not limited to simple addition, but also possible in other tasks, such as single-digit arithmetic WPs. Fuchs et al. (2009) investigated so called “Number combination.” This means simple arithmetic problems that can be solved via counting or decomposition strategies or committed to long term memory for automatic retrieval. Here, arithmetic fact retrieval had to be differentiated from other strategies on three levels: operational, items difficulty, and individual differences. These numerical factors influence solution strategies in arithmetic and WPs as well. Decomposition and counting require more working memory and therefore leave less resources for the built-up and maintenance of a text situations model. However, both individual and stimulus differences should also be considered. For instance, Grabner et al. (2009) showed in an fMRI study that not only problem but also individual strategy choice contributed to fact retrieval processes when solving multiplications.

### Information Relevance and Step-Wise Problem Processing

One relatively extensively studied factor in WPs is the relevance of the information. Individuals have to extract the relevant information from the text in order to carry out the correct solution. Secondary information distracts people from recognizing the underlying mathematical relations (Schley and Fujita, 2014). This extra information may also be presented in the form of an extra number or an extra operational step – one-step (i.e., one calculation step has to be performed) and two-step problems (i.e., two calculation steps have to be performed). Problem complexity increases with the addition of steps (Terao et al., 2004), as well as the addition of irrelevant information to the problem (Kingsdorf and Krawec, 2014) Presence of extraneous information and the need for an extra step reduced the accuracy of the students’ solutions, because students believe that all of the numbers in a WP should be used. All other factors being kept constant, two-step problems are much more error-prone than one-step problems (Muth, 1992). However, it cannot be concluded that the reason for two-step problems being more difficult is arithmetic complexity, because in two-step problems, the WP has also become more difficult linguistically as it usually contains more phrases and semantic distractors.

### Other Numerical Processes and Representation

Several other numerical processes and representations have not been investigated in WPs. For instance, as shortly outlined above, one major factor in simple calculation problems, which can be studied in isolation, is the presence or absence of a carry operation. Children and adults take longer and commit more errors when computing the solution to a sum for which adding the units leads to a change in the number of 10s (e.g., 14 + 9 = 23; Furst and Hitch, 2000; Deschuyteneer et al., 2005) than when it does not (e.g., 11 + 12 = 23). This effect is known as the carry effect; in carry problems, a one needs to be carried from the unit slot to the decade slot. The carry effect is influenced by various processes, but even by language structure (Goebel et al., 2014). Language influences on the difficulty of the numerical computations within a WP have to our knowledge not been studied. Other central topics of numerical cognition such as, e.g., number and symbol sense contribute to WP solving are also open questions (MacGregor and Price, 1999). We have chosen some selected variables/factors, which have been investigated in the WP research.

## Connecting Linguistic and Mathematical Factors

There are so many linguistic influences on numerical cognition and arithmetic that this justifies a special issue like this. For instance, number word structure seems to play an essential role. Children growing up with regular number word structure usually perform better in variety of numerical tasks from basic verbal counting up to arithmetic, e.g., Miller et al. (1995) or Dowker et al. (2008). In addition, the consistency of the order of the number word system and the Arabic number influences transcoding (Zuber et al., 2009; Pixner et al., 2011a; Imbo et al., 2014) number comparison (Nuerk et al., 2005; Pixner et al., 2011b; Klein et al., 2013; Moeller et al., 2014) calculation (Goebel et al., 2014); see also (Brysbaert et al., 1998; Colomé et al., 2010). In addition, reading direction influences numerical processes like the SNARC effect (Shaki et al., 2009; Fischer and Shaki, 2014); see Goebel et al. (2011) for reviews. Finally, grammatical and syntactic properties of elementary number words influence early number acquisition (Sarnecka, 2013) and spatial-numerical representations (Roettger and Domahs, 2015). The linguistic influence on numerical cognition is hardly debatable any more. In fact, Lachmair et al. (2014) argue for a connection of language and words, O´Neill (2013) states that the link between language and mathematics might originate from the same roots, and “required abilities are not that split up as we think,” and MacGregor and Price (1999) also argue that between language and mathematics in WPs there is deep connection: “that the cognitive ability that drives symbol processing is the connection between language and maths.” Nevertheless, systematic variation of both linguistic and numerical factors in WPs is scarce – though Bassok et al. (1998) already found that semantic relations between objects in the text of mathematical WPs were highly positively correlated with arithmetic operations that took these objects as arguments. Neural correlates of visualization and verbalization during arithmetic WP study also suggest that mental arithmetic in WPs is influenced by language processing (Zarnhofer et al., 2013).

Word problems require some connection between linguistic and mathematical understanding by the very nature of the task, because at least children do not have a repertoire of “highly automatized schemata” for representing the different problem types (Garcia et al., 2006). Therefore, it is not surprising that children make more errors when solving WPs compared to number problems (Geary, 1996; Koedinger and Nathan, 2004). Children are able to solve several types of addition and subtraction problems before they start formal schooling (De Corte and Verschaffel, 1987; Lean et al., 1990), and understand numerical concepts before seeing WPs in their curricula (Garcia et al., 2006). Therefore, most studies implicitly assume that problem solvers always have the necessary basic arithmetic skills, even in the case of children. This may lead to the misconception that numbers may play a lesser role than they actually do and factors other than computational skills are a major source of difficulty with WPs (Nesher, 1976; Reusser, 1993). In this aspect, it is also important to note that difficulties in solving WPs have been reported that could be neither attributed to the lack of general reading comprehension skills nor to the lack of general mathematical skills (Hegarty et al., 1995). Nevertheless, linguistics and numerical factors are usually not independently manipulated in WPs and not even dissociated by other means (e.g., regressions). What is more, their interaction is rarely studied [for an exception, see Verschaffel and De Corte (1990)].

### Lexical Consistency Effect

One of the few frequently studied factors examining the relation between text and arithmetic problems is lexical inconsistency. Some WPs contain linguistic markers as “less” or “more.” In the direct translation strategy (Hegarty et al., 1995) students simply associate “less” with subtraction and “more” with addition. They search for linguistic markers and keywords. In the problem model strategy, they construct a mental model of the problem and plan their solution on the basis of this model. Successful learners are more likely to employ the problem model strategy; they focus more on variables names and relational terms and successful problem solvers re-read the text less frequently (Pape, 2003) in the eye-tracking studies. Unsuccessful learners, on the other hand, seem to rely on the direct translation strategy; they focus on numerals and on relational terms, and linguistics markedness in the (Hegarty et al., 1992) eyetracking study. This leads to wrong solutions in lexically inconsistent texts, where “more” is associated with subtraction and “less” with addition. To give an example for lexical inconsistency, consider the following WP adapted from Boonen et al. (2013) “At the grocery store, a bottle of olive oil costs 7 check veera. That is 2 check veera more than at the supermarket. How much will [a bottle of olive oil] cost in the supermarket?” The anticipated difficulty in comprehension and finding the correct solution is due to the fact that the adverb “more” evokes the concept of addition, but the correct solution is not 7 + 2 but 7 - 2, given the way the text is organized. Verschaffel et al. (1992) found such a reaction time consistency effect for children but not for adults. Nesher (1976) and Lean et al. (1990) obtained similar results in experiments with groups of non-disadvantaged children and students, showing that linguistic semantic consistency with respect to the required mathematical operation is an important determinant of task difficulty. Inconsistent language results in a high error rate and longer response time (Hegarty et al., 1992), even in Verschaffel (1994) retelling one-step compared WPs showed a strong evidence for the consistency hypothesis. Students made ∼13% more reversal errors on inconsistent than on consistent language problems and the difficulty of comprehending inconsistent-language problems were increased when the correct arithmetic operation was an increase. However, the literature is inconsistent if the consistency effect is present in both students and children. Children find it easier to convert the relation term “more than” into subtraction operation than the relational term “less than” into an addition operation (Lewis and Mayer, 1987; Verschaffel et al., 1992; Pape, 2003; van der Schoot et al., 2009).

When neither reading comprehension nor arithmetic skills alone can explain failure to solve WPs, a possible explanation is that linguistic complexity and numerical complexity rely on the same resources (e.g., working memory). The premise is that there is not an absolute atomic concept of difficulty for WPs. Rather; there are multiple linguistic and numerical factors which contribute to a problem’s complexity. It is a combination of these factors that might make a problem additively more or less difficult because they exert demands on more general resources like working memory. Generally, problem solving performance is related to the ability of reducing the accessibility of no target and irrelevant information in the memory (Passolunghi and Siegel, 2001). Working memory contributes to early arithmetic performance, and studies also show that this extends to WP solving (Lee et al., 2004) due to semantic memory representation “less than” which is more complex than “more than.” Changes in the structure of the text has more demand on the working. It has been suggested that WPs in general are related to working memory (Swanson et al., 1993). This will probably also be influenced by instruction specifying how participants have to solve a WP, and the method of evaluation, and scoring system. In Van Dijk and Kintsch’s (1983) model of reading comprehension, working memory is used to keep a number of text propositions active simultaneously. In particular, working memory has been related to each single component mentioned above, such as text-problem relation, the linguistic complexity, and the arithmetic complexity.

## Future Direction, Open Questions

Word problem difficulty is influenced by the complexity of linguistic factors, numerical factors, and their interrelation. To better understand the difficulty of WPs, it would be desirable to manipulate such variables and their interaction following the principle of isolated variation. To support a systematic investigation, the variables to be manipulated also need to be discussed against the backdrop of the relevant conceptual and empirical issues in the underlying fields, linguistics, and numerical cognition. This has too rarely been the case in the past. For instance, in the earlier studies on algebra WPs, the linguistic cues are of mixed categories (adverbs, verbs, nouns, etc.) and the effect of the complexity of syntactic structures is not taken into account. Similarly, numerical complexity like basic number properties (e.g., magnitude, place-value processing for multi-digit numbers) or the complexity of underlying arithmetic computations (e.g., carry effects for addition, relatedness, or consistency effects for multiplication) are often neglected. WP research would be well advised to take into account the foundational categories, properties and findings of both numerical cognition and linguistics when it examines which WPs are difficult for which groups and why. Not only the main effects of numerical and linguistic complexity should be studied, but also their interaction. To make the relevant aspects explicit, **Figure 1** sketches an overall process model of WP solving.

The joint investigation of linguistic and numerical processes also needs to take into account joint moderator variables such as working memory in order to explore the possible interactions between them. Since working memory affects all components of complexity of a WP, the difficulties triggered may not be simply additive, but also interactive. The resolution of linguistic and numerical difficulties may rely on the same processing stages and resources (Sternberg, 1969). To investigate this, more collaboration between linguists and numerical cognition researchers would be desirable.

Finally, we suggest a differential-psychological approach to WP research. Different students may have a problem with different types of WPs. Linguistically rather weak students may have problems with linguistically complex WPs, and arithmetically rather weak students with arithmetically complex problems. Undifferentiated presentation of WPs in experiments will not provide sufficient information about which skills and processes an individual child should practice. Only with such differentiation on an item level (as regards linguistic and numerical complexity and their interrelation) and on an individual level (as regards linguistic and numerical skills and general cognitive abilities) will it be possible to understand why a particular child has its individual difficulties with particular WP types. Such an understanding, however, is essential to promote tailored learning of one of the most difficult arithmetic problem types that students encounter in school.

## Conflict of Interest Statement

The authors declare that the research was conducted in the absence of any commercial or financial relationships that could be construed as a potential conflict of interest.
